# Exploring the Causal Effect of Mitochondrial DNA Copy Number on Obstructive Sleep Apnea

**DOI:** 10.1002/brb3.70720

**Published:** 2025-08-25

**Authors:** Ping Ji, Yujiang Fan, Junling Li, Zhaoan Deng, Guofu Zhang, Jianbin Du

**Affiliations:** ^1^ Department of Geriatric Psychiatry, The Affiliated Mental Health Center of Jiangnan University Wuxi Central Rehabilitation Hospital Wuxi China; ^2^ Department of Mental Health People's Hospital of Huantai County Zibo China; ^3^ Department of Psychiatry, Wuxi Huishan District People's Hospital Affiliated Huishan Hospital of Xinglin College Nantong University Wuxi China

**Keywords:** immune cell, mendelian randomization analysis, mitochondrial DNA, mtDNA copy number, obstructive sleep apnea, psychiatry

## Abstract

**Purpose:**

Although some studies have established a clear association between mitochondrial DNA (mtDNA) copy number and obstructive sleep apnea (OSA), the causative relationship between the two remains unclear, which is what this study aims to explore.

**Method:**

We investigated the causal relationship between mtDNA copy number and OSA based on public genome‐wide association study data utilizing a two‐sample Mendelian randomization (MR) analysis and also explored the mediating role of immune cells between the two using a mediator MR analysis. We estimated the causal effects primarily using the inverse variance weighted method and conducted sensitivity analyses based on the MR‐Egger intercept method and Cochran's *Q* test.

**Finding:**

We found that mtDNA copy number had a significant negative causal effect on OSA (odd ratio [OR] [95% confidence interval (CI)] = 0.844 [0.778‐0.911], *p* = 0.03), whereas OSA did not have a causal effect on mtDNA copy number (OR [95% CI] = 0.995 [0.979–1.01], *p* = 0.791). We identified that the terminally differentiated CD4‐CD8^−^ T cell Absolute Count met the requirements for mediation analysis (OR [95% CI]_as exposure_ = 0.989 [0.985–0.993], *p* = 0.016, OR[95% CI]_as outcome =_ 0.717 [0.529–0.973], *p* = 0.033). Under this condition, the mediation effect size of the immune cell was 0.003, which is considered to have no mediating effect either using the bootstrap method or the two‐step method (*p* > 0.05).

**Conclusion:**

Our study indicates that reducing mtDNA copy numbers is a risk factor for the development of OSA, rather than a consequence of it. Improving mitochondrial dysfunction may help prevent or treat OSA.

## Graphical abstract

Mitochondrial DNA (mtDNA) copy number may act as a biological marker and therapeutic target for obstructive sleep apnea (OSA). Maintenance of normal mtDNA copy numbers can prevent the development of OSA, and antioxidant therapy should be an important intervention against OSA in the future.

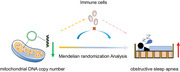

## 1 Introduction

Obstructive sleep apnea (OSA) syndrome is a prevalent sleep disorder characterized by recurrent episodes of apnea and hypoventilation during sleep, resulting in intermittent hypoxemia and disrupted sleep architecture (Sanz‐Rubio et al. 2020). These apneic events lead to frequent awakenings throughout the night, reduced sleep quality, excessive daytime sleepiness, and a range of associated physical and psychological issues (L. Xu et al. 2020). Chronic OSA is linked to numerous health complications, including cardiovascular diseases (such as hypertension, heart disease, and stroke) (Turnbull et al. 2019; Javaheri et al. 2016; Lipford et al. 2015), metabolic syndrome (including diabetes mellitus) (Chopra et al. 2017), and cognitive impairment (Chou et al. 2013). Additionally, individuals with OSA may face an elevated risk of traffic accidents due to daytime drowsiness (F. Song et al. 2020). However, the pathogenesis of OSA is multifactorial, involving anatomical abnormalities, physiological dysregulation, and neuromuscular dysfunction (Remmers 1984; Bradley and Phillipson 1985; White 2005). Anatomic factors such as large neck circumference, soft tissue, bone, or vessel abnormalities can contribute to increased pressure around the upper airway, leading to collapsibility (Wondie et al. 2022). Research indicates that obesity, male gender, and narrowed airways are significant risk factors for OSA, while age, family history, smoking, alcohol consumption, hypertension, and diabetes also increase the risk of developing the condition (Mitra et al. 2021; Franklin and Lindberg 2015; Parish and Somers 2004). Recent studies on the interaction between mitochondrial oxidative stress and immune responses in OSA reflect the complexity of the pathogenesis of this disease (Jelic et al. 2008).

Mitochondrial DNA (mtDNA) copy number refers to the quantity of mitochondrial DNA present within a cell. Mitochondria, often described as the cell's energy factories, possess their own DNA that encodes key proteins involved in oxidative phosphorylation—an essential process for cellular respiration and energy production (Baechler et al. 2019). The mtDNA copy number reflect the status of mitochondrial function, and its quantity has an impact on cellular health and the overall well‐being of the organism (Kinghorn et al. 2015). In conditions of severe oxidative stress, when oxidative damage exceeds the capacity of the mtDNA repair system, mtDNA sustains significant damage and may undergo extensive mutations (Wu et al. 2019). This activates the mitochondrial autophagy mechanism, which removes the dysfunctional mitochondria, ultimately leading to a reduction in mtDNA copy number. Research has demonstrated that a reduction in mtDNA copy number is associated with various conditions, including Parkinson's disease (Pyle et al. 2016), schizophrenia (Shivakumar et al. 2020), and other neurodegenerative disorders (Klein et al. 2021), as well as aging (Foote et al. 2018), diabetes (Memon et al. 2022), cardiovascular diseases (Ashar et al. 2017), and a range of cancers (Meng et al. 2016).

mtDNA copy number has also been linked to sleep, and individuals with poor sleep quality tend to be accompanied by reduced mtDNA copy numbers (Han et al. 2024). Research indicates that sleep has multiple positive effects on mitochondrial health and function, including promoting mitochondrial repair and renewal (Mauri et al. 2022) and reducing dysfunction and mtDNA damage (Richardson and Mailloux 2023; Xie et al. 2013). Therefore, getting adequate sleep is critical for maintaining mitochondrial health and overall health. Moreover, numerous polymorphic mutation sites have been identified in the D‐loop region of mtDNA in obstructive sleep apnea‐hypopnea syndrome (OSAHS) patients, suggesting a possible connection to the pathophysiological processes underlying OSAHS (Huang et al. 2014). In OSA, intermittent hypoxia (IH) represents a key pathophysiologic feature that may contribute to neuronal cell death and neurocognitive deficits (Y. Wang et al. 2019). Research has demonstrated that the cytoplasmic release of mtDNA in response to IH can activate the cyclic GMP‐AMP synthase (cGAS)‐stimulator of interferon genes (STING) pathway—a trigger for immune responses and neuronal death in degenerative diseases, leading to neuronal apoptosis via the regulation of endoplasmic reticulum stress (S. Wang et al. 2024). In addition, chronic IH and sleep fragmentation are powerful drivers of immune system activation and dysregulation, inducing activation of neutrophils, monocytes, and macrophages, leading to increased production of reactive oxygen species (ROS) and pro‐inflammatory cytokines (J. Xu et al. 2022). Activation of adaptive immune function leads to imbalances in T‐cell subsets, including increased Th17 responses and decreased regulatory T‐cell activity, which may perpetuate the inflammatory process (Kirschenbaum and Flanery 1983). Furthermore, chronic IH and oxidative stress impair immune cell mitochondrial function, further exacerbating immune dysregulation. Mitochondrial dysfunction amplifies oxidative stress, which activates inflammatory signaling pathways, while immune dysregulation exacerbates mitochondrial damage through cytokine‐induced oxidative stress and metabolic disturbances. The interaction between mitochondrial dysfunction and immune dysregulation creates a vicious cycle that exacerbates the pathophysiology and systemic comorbidities of OSA (Kim et al. 2014).

Collectively, these studies suggest that mitochondrial dysfunction and immune‐inflammatory responses are involved in the onset and progression of OSA. However, further in‐depth randomized studies are needed to establish causative relationships, and a comprehensive understanding of these underlying mechanisms will facilitate the development of more effective prevention and treatment strategies for OSA.

Mendelian randomization (MR) is an epidemiological method that leverages genetic variation as an instrumental variable to assess causal relationships. At its core, MR uses genetic variants (single‐nucleotide polymorphisms, SNPs) to emulate the effects of randomized controlled trials (RCTs), allowing researchers to investigate causal links between an exposure (e.g., lifestyle or environmental factors) and a disease outcome (Gill et al. 2020). In recent years, MR has been widely applied in studies examining both somatic and psychiatric disorders (Du, Baranova, et al. 2024; Du, Fang, et al. 2024; Zhang et al. 2022). To further deepen our understanding of the roles of oxidative stress and immune response in the pathogenesis of OSA, we discovered and discussed the causative relationship between mtDNA copy number and OSA using an MR approach based on publicly available genome‐wide association study (GWAS) data, and ruling out the mediating role of immune cells between the two.

## 2 Materials and Methods

### 2.1 Overall Study Design

**FIGURE 1:**
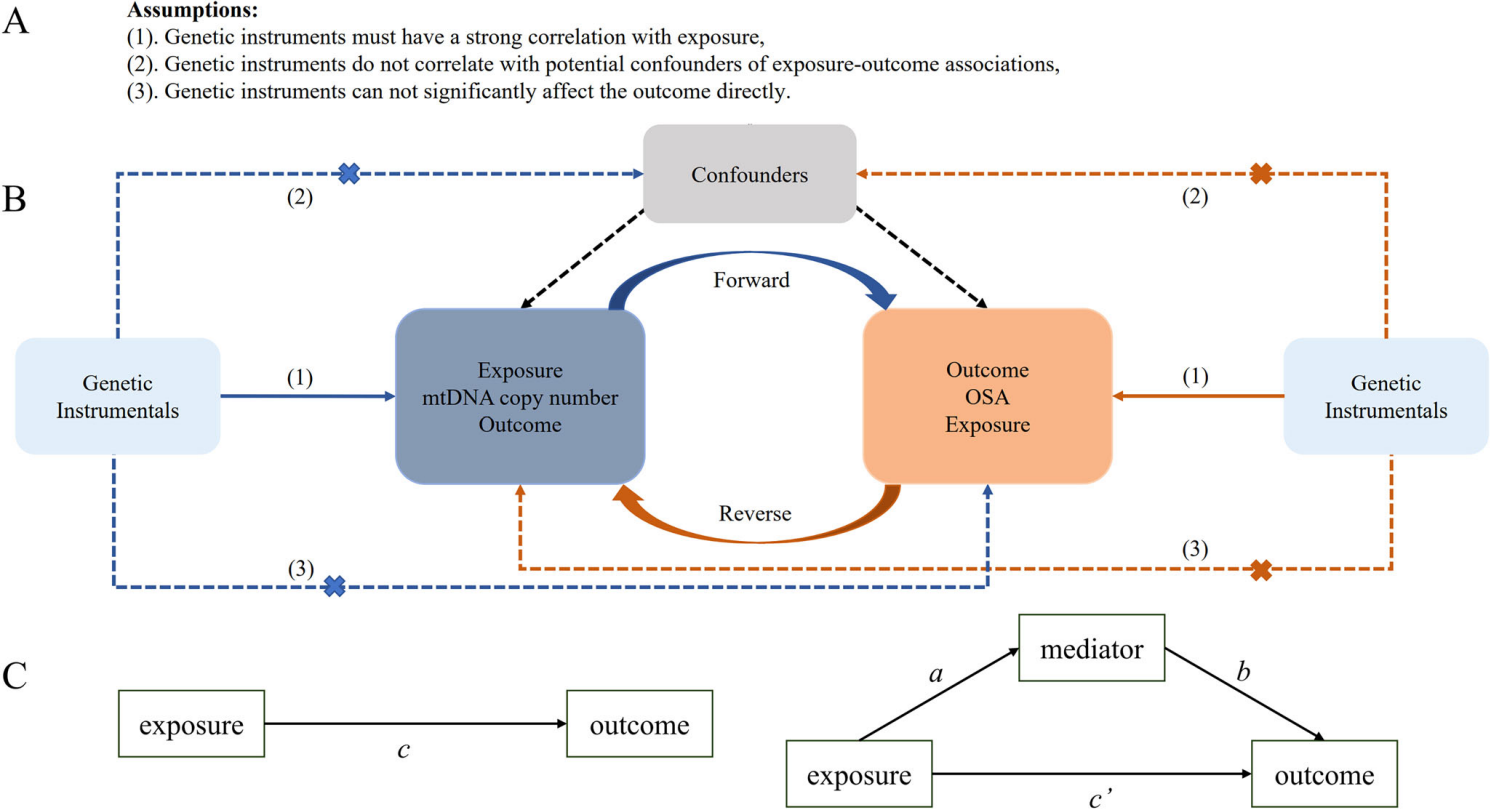
Schematic and design for bidirectional MR and mediation analyses. (A) The three core assumptions of Mendelian randomization analysis. (B) The analytical flow of bidirectional Mendelian randomization. Dark blue represents forward MR analyses, and dark brown represents reverse MR analyses. (C) Schematic diagram of mediation analysis. *c* represents the total effect of exposure on the outcome, and *a*, *b*, and *c’* represent the direct effect of exposure on the mediator, mediator on the outcome, and exposure on the outcome, respectively. OSA: obstructive sleep apnea.

This study consisted of two components: a two‐sample MR analysis and a mediator MR analysis. In the two‐sample MR analysis, mtDNA copy number and OSA were evaluated as exposures and outcomes in both forward and reverse MR assessments. Previous studies have indicated that oxidative stress interacts synergistically with the immune response to influence OSA. Based on this, we selected immune cell traits related to immune responses as mediator variables for the mediation MR analysis. In principle, immune cell traits influenced by mtDNA copy number and showing significant causal effects on OSA can be considered potential mediators. To identify these candidates, we first conducted a two‐sample MR analysis using immune cell traits as the exposure and OSA as the outcome, collecting immune cells with significant causal effects on OSA. Next, we performed an MR analysis with mtDNA copy number as the exposure and the previously identified immune cell traits as the outcome to identify immune cells with positive causal associations. Finally, we analyzed the mediating role of these immune cell traits in the relationship between mtDNA copy number and OSA. The selection of instrumental variables (IVs) was guided by the three core principles of MR research, as illustrated in Figure 1A,B.

### 2.2 GWAS Data Sources

MR analysis was conducted using publicly available GWAS summary statistics for OSA from the FinnGen consortium, which included 33,423 cases and 336,659 controls (Kurki et al. 2023). The diagnosis of OSA was established in accordance with the International Classification of Diseases (ICD‐10: G47.3, ICD‐9: 347.2A), based on patients' reported symptoms, clinical examinations, and sleep assessments utilizing the apnea‐hypopnea index or a respiratory events index of five events per hour. Factors such as age, sex, genotyping microarray, genetic relatedness, and the first 10 principal components were considered in the analysis. Data on mtDNA copy number, encompassing 383,476 samples, were obtained from the UK Biobank (Chong et al. 2022). Immune cell traits data were sourced from a cohort of 3,757 individuals (Huang et al. 2014). The ethical approval procedures for the original study included in the GWAS are detailed in the respective publication. Each cohort in the GWAS study obtained ethical approval and participation consent, and summary‐level data were publicly released.

### 2.3 Selection of IVs

We selected SNPs with high statistical significance (1 × 10^−5^ for mtDNA copy number and immune cell traits, 5 × 10^−8^ for OSA) as potential IVs based on the principles of MR analysis. Reliable SNPs were selected through linkage disequilibrium clumping (*r* ^2^ threshold < 0.001 within a 10,000 kb distance) based on the European 1000 Genomes reference panel. To satisfy the first assumption of MR and to estimate weak instrument bias and instrument strength, we calculated *F*‐statistics (*β* ^2^/SE^2^), recommending F‐statistics > 10 for further MR analysis.

### 2.4 Two Sample MR Analyses

Three distinct methods, each offering unique strengths, were employed to estimate the causal association between exposure and outcome. (i) The random effects inverse variance weighting (IVW) approach was selected as the preferred method due to its ability to robustly estimate causality even in the presence of heterogeneity in IVs (Mounier and Kutalik 2023). (ii) MR‐Egger is a method that accommodates pleiotropy, allowing IVs to influence the outcome through pathways other than exposure‐related instruments (Burgess and Thompson 2017). (iii) Weighted median method also can provides a robust causal estimate even when up to 50% of the IVs are invalid. MR‐Egger intercepts are employed to assess the presence of horizontal pleiotropy in IVs and heterogeneity was evaluated using Cochran's *Q* test, with *p*‐value < 0.05 indicating their presence. The leave‐one‐out method further explores the impact of heterogeneity, providing valuable insights into the potential influence of individual IVs on estimating causal effects and assessing the robustness of the results.

### 2.5 Mediation MR Analyses

The mediated MR method was carried out on the basis of the two‐sample MR method. Briefly, immune cell traits and OSA were used as exposure and outcome, respectively, to obtain immune cell traits after primary screening. Afterward, mtDNA and immune cell traits were used as exposure and outcome, respectively, to obtain immune cell traits after rescreening again, and this was used as a potential mediating variable. In this context, we defined *a*, *b*, and *c* as representing the direct effect of exposure on the mediator, the mediator on the outcome, and the exposure on the outcome, respectively. The relationship between the three is shown in Figure 1C. The significance of *c* serves as a prerequisite for assessing the indirect effect. The presence of a significant indirect effect is indicated when both *a* and *b* are significant. If *c'* (i.e., *c* − *a × b*) remains significant, it signifies the presence of a significant direct effect, suggesting a partial mediating effect. Conversely, if *c'* is not significant, it indicates a full mediating effect. To assess the significance of the indirect effect, we employed the two‐step method as recommended by Hayes (Hayes and Rockwood 2017). We conducted 5000 bootstrap iterations with a 95% confidence interval (CI). If zero does not fall within the 95% CI of the bootstrap results for the indirect effect, it implies that the indirect effect significantly deviates from zero, providing evidence for the existence of a mediating effect.

### 2.6 Statistical Methods

The MR analysis in this study utilized the TwoSampleMR (Hemani et al. 2018) and RMediation (Tofighi and MacKinnon 2011) package based on the R language. It was considered statistically significant when the *p*‐value was < 0.05. All *p* values are two‐tailed in this study.

## 3 Results

### 3.1 Causal Effect of mtDNA Copy Number on OSA

**FIGURE 2:**
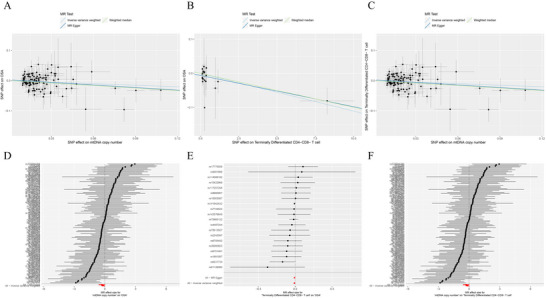
Visualization of causal effects across exposure and outcome conditions. Scatter plots reflect causal effect size and direction. Forest plots reflect the effect sizes of each instrumental variable. (A and D), (B and E), and (C and F) represent the results of causative relationships between mtDNA copy number and OSA, terminally differentiated CD4‐CD8^−^ T cell and OSA, and mtDNA copy number and terminally differentiated CD4‐CD8^−^ T cell, respectively.

We found a significant negative causative effect of mtDNA copy number on OSA (odd ratio [OR] [95% CI] = 0.844 [0.778‐0.911], *p* = 0.03). This implies that maintaining a certain level of mtDNA copy number is protective against OSA. The MR‐Egger intercept and MR‐PRESSO global tests ruled out the possibility of horizontal pleiotropy, and Cochran's *Q* tests for IVW and MR‐Egger methods showed no heterogeneity in the IVs (Table 1). Scatter plots showed the direction and stability of causal effects (Figure 2A). However, we did not find a significant causative effect of OSA on mtDNA copy number (OR [95% CI] = 0.995 [0.979‐1.01], *p* = 0.791). Details can be found in Table 1.

**TABLE 1:** Significant MR analysis results in the discovery samples.

Exposure	Outcome	Method	N_SNPs	*b* (SE)	OR [95% CI]	*p*
mtDNA copy number	OSA	IVW	122	−0.169 (0.078)	0.844[0.778–0.911]	0.03
		WM	122	−0.274 (0.116)	0.76[0.662–0.859]	0.018
		MR‐Egger	122	−0283 (0.162)	0.754[0.615–0.893]	0.083
OSA	mtDNA copy number	IVW	5	−0.005 (0.018)	0.995[0.979–1.01]	0.791
		WM	5	−0.009 (0.017)	0.991[0.977–1.005]	0.601
		MR‐Egger	5	0.143 (0.069)	1.153[1.073–1.234]	0.13
mtDNA copy number	CD4‐CD8^−^ T cell Absolute Count	IVW	136	−0.332(0.155)	0.717(0.529–0.973)	0.033
		WM	136	−0.179(0.331)	0.835(0.436–1.598)	0.588
		MR‐Egger	136	−0.272(0.225)	0.761(0.489–1.184)	0.226
CD4‐CD8^−^ T cell Absolute Count	OSA	IVW	20	−0.011(0.005)	0.989[0.985–0.993]	0.016
		WM	20	−0.009(0.005)	0.99[0.985–0.995]	0.075
		MR‐Egger	20	−0.009(0.005)	0.991[0.987–0.995]	0.072

Abbreviations: CI: confidence interval; IVW, inverse variance weighting; MR, mendelian randomization; N_SNP, numbers of single nucleotide polymorphism; OR, odd ratio; OSA, obstructive sleep apnea; WM, weighted mendian.

### 3.2 Causal Effect of Immune Cell on OSA

We found a total of 34 immune cell traits that had a causal effect on OSA at nominal significance. Among them, 16 had positive causal effects (OR: 1.0–1.04) and 18 had negative causal effects (OR: 0.95–0.99). These immune cell traits will serve as potential mediators into the subsequent mediation analysis. Meanwhile, we also found that OSA had nominally significant causal effects on 29 immune cell traits, including 13 positive causal effects (OR: 1.34∼2.02) and 16 negative causal effects (OR: 0.38∼0.75). More details can be found in Tables S1 and S2.

### 3.3 MR Study Results Between mtDNA Copy Number and OSA Mediated by Immune Cells

We again used mtDNA copy number and immune cell traits as exposure and outcome, respectively, and finally screened terminally differentiated CD4‐CD8^−^ T cell Absolute Count as a candidate mediator (Table 2; Figure 2B,C). Under this condition, the total casual effect of mtDNA copy number on OSA was −0.169, the direct effect was −0.173, and the effect size of the immune cell in this process was 0.003. However, the immune cell was considered to have no mediating effect either using the bootstrap method or the two‐step method (*p* > 0.05). Detailed results are shown in Table 3.

**TABLE 2:** Sensitivity analysis of the effects of immune cell traits on schizophrenia.

			MR‐egger regression		Heterogeneity analyses		
Exposure	Outcome	N_SNPs	Intercept	*p*	Method	*Q*	*Q*‐pval
mtDNA copy number	OSA	122	0.003	0.425	IVW	136.8	0.155
					MR‐Egger	136.1	0.149
mtDNA copy number	CD4‐CD8^−^ T cell Absolute Count	136	0.004	0.604	IVW	153.819	0.128
					MR‐Egger	153.509	0.119
CD4‐CD8^−^ T cell Absolute Count	OSA	20	−0.006	0.177	IVW	11.33	0.9123
					MR‐Egger	9.357	0.951

Abbreviations: IVW, inverse variance weighting; MR, mendelian randomization; N_SNP, numbers of single nucleotide polymorphism; OSA, obstructive sleep apnea.

**TABLE 3:** Significant MR analysis results causally connecting schizophrenia and blood cell counts.

			Bootstrap method		Delta method		
	Effect	SE	Bias‐corrected 95% CI	Percentile 95% CI	SE	95% CI	*p*
Total effect	−0.169	0.084	−0.336 ∼ −0.003	−0.334 ∼ −0.001	—	—	—
Direct effect	−0.173	0.085	−0.337 ∼ −0.002	−0.341 ∼ −0.007	—	—	—
Indirect effect	0.003	0.006	−0.003 ∼ 0.012	0.001 ∼ 0.024	0.052	−0.097 ∼ 0.105	0.943

Abbreviations: CI: confidence interval; SE, standard error.

## 4 Discussion

Our study found that mtDNA copy number has a unidirectional negative causal effect on OSA, and this effect is also not achieved by modulating immune cells. Previous studies have shown that mitochondrial dysfunction is closely associated with the onset and severity of OSA (Edwards et al. 2025). The mtDNA copy numbers can indirectly reflect the quantity and functional state of mitochondria (Tai et al. 2022). Our study found that OSA does not have a direct causal effect on mtDNA copy number, suggesting that the factors causing mitochondrial dysfunction in OSA may not directly originate from mtDNA copy number. However, we also found that mtDNA copy number has a protective effect against OSA, meaning that lower mtDNA copy number may indicate impaired mitochondrial function, leading to reduced cellular tolerance to hypoxia and thereby increasing the risk of OSA.

OSA is a sleep disorder characterized by repeated interruptions in breathing during sleep, which can lead to various health issues, including cardiovascular problems and metabolic disorders. Recent studies have suggested a potential link between OSA and mtDNA copy number, as the stress of IH may influence mitochondrial function and replication (Choi et al. 2016). Immune cells play a crucial role in the body's response to the inflammation and oxidative stress associated with OSA, potentially affecting mtDNA dynamics (Badran et al. 2019). The mechanisms underlying these interactions may involve altered immune responses and mitochondrial dysfunction, which can exacerbate the effects of OSA on overall health. Causality remains a complex issue, as while there is evidence suggesting that OSA can impact mtDNA copy number and immune cell function, the reverse relationship and other confounding factors must also be considered. Understanding these interactions is essential for developing targeted therapies and interventions for individuals suffering from sleep apnea. Further research is needed to clarify the precise mechanisms and establish a definitive causal relationship between these elements.

OSA involves mechanisms such as IH, which can lead to cognitive impairment and myocardial injury through pyroptosis, while also affecting circadian rhythms and inflammatory responses, underscoring its complex interplay with comorbidities and the necessity for targeted treatments (Manolis et al. 2025; Wei et al. 2024; Li et al. 2024). OSA is influenced by mechanisms including weight loss, enhanced insulin sensitivity, and decreased upper airway fat through glucagon‐like peptide‐1 receptor agonists (GLP‐1RA), resulting in significant reductions in the apnea‐hypopnea index (AHI) and improved sleep quality (Dragonieri et al. 2024). Additionally, increased mtDNA copy number following melatonin treatment has been shown to alleviate oxidative stress and activate the PGC‐1α/TFAM pathway, indicating a mechanism by which melatonin may enhance mitochondrial function, particularly in middle‐aged mice with sarcopenia (Fang et al. 2025). Furthermore, mtDNA copy number plays a dual role in various mechanisms: it positively influences mitochondrial function by enhancing respiration and ATP production when inhibited by miR‐146a‐5p (Krishnan et al. 2024), while also negatively impacting ethanol‐induced brain injury and sperm epigenetic aging (Togre et al. 2024; Amir et al. 2024). This intricate relationship between OSA, mtDNA copy number, and their respective mechanisms highlights the potential for targeted therapeutic strategies to mitigate the adverse effects of OSA.

The interplay between mtDNA copy number and immune cell function is critical in understanding the broader implications of OSAS. Increased mtDNA copy number has been linked to enhanced mitochondrial function and protection against oxidative stress, which may be particularly relevant in the context of OSA‐related hypoxia (Fang et al. 2025; J. Song et al. 2024). Causality is underscored by findings that reduced mtDNA copy numbers can accelerate reproductive aging under oxidative stress conditions (Long et al. 2024). Furthermore, immune cells exhibit dual roles in various pathological states, influencing both tumor immunity and inflammatory responses (Cao et al. 2024; Cao and Liu 2024). The regulation of pro‐inflammatory responses by immune cells, such as through NOD2, highlights the complex mechanisms at play in conditions like OSA, where immune dysregulation may contribute to the disease's systemic effects (Masaki et al. 2024). A bidirectional causative relationship between immune cells and OSA was also found in this study, suggesting a more complex role of immune function in the progression of OSA. Considering that OSA is also a low‐grade chronic respiratory inflammation, the recurrent chronic IH during sleep triggers an anti‐inflammatory cascade response, and it becomes easy to understand that it causes alterations in different directions on different immune cell types. For example, it has been found that all subpopulations of dendritic cells are significantly reduced in the peripheral blood of OSA patients, with the myeloid dendritic cell (mDC) and plasmacytoid dendritic cell (pDC) subpopulations being more severely impaired (Galati et al. 2020). Of course, due to the complexity of immune function, results regarding changes in immune cell types in the disease are not consistent (Domagała‐Kulawik et al. 2015; Gaoatswe et al. 2015). Regardless, our results rule out a regulatory role for immune cells in it, highlighting the direct role and importance of mtDNA copy number on OSA.

This study has several limitations. First, the samples analyzed were exclusively of European origin, which limits the generalizability of the findings to other populations due to genetic variability across different ethnic groups. Second, while exploring the causal effects of mtDNA copy number and OSA, immune cell types were analyzed based on nominal significance. This approach, aimed at minimizing type 2 errors, may have inadvertently increased the likelihood of false positives. Third, while oxidative stress appears to have a causal effect on OSA, relying solely on mtDNA copy number as an indicator may provide a relatively weak signal. Further validation is required, combining more extensive genetic data and clinical trials to strengthen the evidence and enhance the robustness of the findings. Lastly, it is important to acknowledge that the relationship between mtDNA copy number and disease may be influenced by various factors, including genetic background, lifestyle, and environmental exposures. While our study identified a causal relationship between mtDNA copy number and OSA from a genetic perspective, it remains essential to comprehensively consider these factors when exploring the effects of mtDNA copy number on disease in greater depth.

## 5 Conclusion

mtDNA copy number may act as a biological marker and therapeutic target for OSA. Reducing mtDNA copy numbers is a risk factor for the development of OSA, rather than a consequence of it. Improving mitochondrial dysfunction may help prevent or treat OSA.

## Author Contributions

**Ping Ji**: writing–original draft, data curation, formal analysis. **Yujiang Fan**: data curation, investigation. **Junling Li**: data curation, investigation. **Zhaoan Deng**: data curation, investigation. **Guofu Zhang**: methodology, validation, supervision, project administration. **Jianbin Du**: methodology, validation, formal analysis, supervision, project administration, writing–original draft.

## Peer Review

The peer review history for this article is available at https://publons.com/publon/10.1002/brb3.70720.

## Supporting information




**Supplementary Table**: brb370720‐sup‐0001‐tableS1.xlsx

**Supplementary Table**: brb370720‐sup‐0002‐tableS2.xlsx

## Data Availability

The GWAS data of OSA for this study from the FinnGen consortium can be found in the FinnGen study platform (https://www.finngen.fi/en/access_results). The GWAS data of mitochondrial DNA copy numbers from the UK Biobank can be downloaded in the GWAS catalog (https://www.ebi.ac.uk/gwas/studies/, accession numbers: GCST 90026372). This study's GWAS data on immune cell traits can be found in the GWAS catalog (https://www.ebi.ac.uk/gwas/studies/; accession numbers: from GCST0001391 to GCST0002121).
